# 3-(2,6-Dioxopiperidin-3-yl)-3-aza­bicyclo­[3.2.0]heptane-2,4-dione

**DOI:** 10.1107/S1600536809002839

**Published:** 2009-01-28

**Authors:** Yousef M. Hijji, Ellis Benjamin, Earl Benjamin, Ray J. Butcher, Jerry P. Jasinski

**Affiliations:** a Department of Chemistry, Morgan State University, Baltimore, MD 21251, USA; b Department of Chemistry and Physics, Arkansas State University, PO Box 419, State University, AR 72467, USA; c Department of Chemistry, Howard University, 525 College Street NW, Washington, DC 20059, USA; d Department of Chemistry, Keene State College, 229 Main Street, Keene, NH 03435-2001, USA

## Abstract

The title mol­ecule, C_11_H_12_N_2_O_4_, consists of a 3-aza­bicyclo­[3.2.0]heptane group containing a nearly planar cyclo­butane ring (r.m.s. deviation of fitted atoms is 0.0609 Å), fused to a pyrrolidine ring, bonded to a 2,6-dioxopiperidine ring at the 3-position. The angle between the mean planes of the cyclo­butane and fused pyrrolidine ring is 67.6 (6)°. The dihedral angles between the mean planes of the pyrrolidine and cyclo­butane rings and the dioxopiperidine ring are 73.9 (2) and 62.4 (4)°, respectively. The pyrrolidine and dioxopiperidine rings are twisted about the 3-yl group [torsion angles = −55.0 (1) and 115.0 (1)°] in a nearly perpendicular manner. Crystal packing is influenced by extensive inter­molecular C—H⋯O and N—H⋯O inter­actions between all four carbonyl O atoms and H atoms from the cyclo­butane and dioxopiperidine rings, as well as between the N atom and an H atom from the cyclo­butane ring. In addition, weak π-ring interactions also occur between H atoms from the cyclobutane ring and the five-membered pyrrolidine ring. As a result, mol­ecules are linked into infinite chains diagonally along the [101] plane of the unit cell in an alternate inverted pattern.

## Related literature

For related structures, see: Muller & Man (2008 ▶); Yamamoto *et al.* (2008 ▶); Zeldis (2008 ▶). For related literature, see: Carson *et al.* (2004 ▶); Werbel *et al.* (1968 ▶); Cremer & Pople (1975 ▶); Schmidt & Polik (2007 ▶).
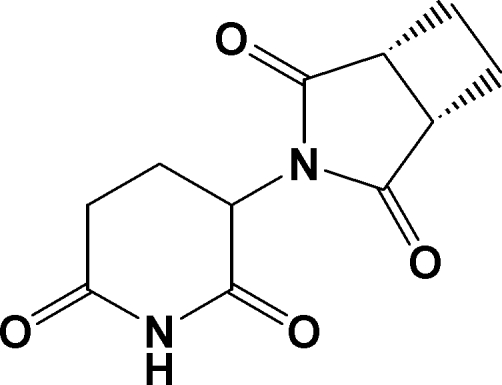


## Experimental

### 

#### Crystal data

C_11_H_12_N_2_O_4_
*M* *_r_* = 236.23
Monoclinic, 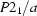

*a* = 10.7332 (7) Å
*b* = 9.9358 (5) Å
*c* = 11.0753 (7) Å
β = 116.201 (8)°
*V* = 1059.75 (13) Å^3^
*Z* = 4
Mo *K*α radiation
μ = 0.11 mm^−1^
*T* = 200 (2) K
0.57 × 0.34 × 0.19 mm

#### Data collection

Oxford Diffraction Gemini diffractometer
Absorption correction: multi-scan (*CrysAlis RED*; Oxford Diffraction, 2007 ▶) *T*_min_ = 0.866, *T*_max_ = 0.975
10798 measured reflections
3496 independent reflections
2193 reflections with *I* > 2σ(*I*)
*R*_int_ = 0.025

#### Refinement

*R*[*F* ^2^ > 2σ(*F* ^2^)] = 0.039
*wR*(*F* ^2^) = 0.108
*S* = 0.99
3496 reflections
154 parameters
H-atom parameters constrained
Δρ_max_ = 0.28 e Å^−3^
Δρ_min_ = −0.25 e Å^−3^

### 

Data collection: *CrysAlisPro* (Oxford Diffraction, 2007 ▶); cell refinement: *CrysAlisPro*; data reduction: *CrysAlis RED*; program(s) used to solve structure: *SHELXS97* (Sheldrick, 2008 ▶); program(s) used to refine structure: *SHELXL97* (Sheldrick, 2008 ▶); molecular graphics: *SHELXTL* (Sheldrick, 2008 ▶); software used to prepare material for publication: *SHELXTL*.

## Supplementary Material

Crystal structure: contains datablocks global, I. DOI: 10.1107/S1600536809002839/cs2103sup1.cif

Structure factors: contains datablocks I. DOI: 10.1107/S1600536809002839/cs2103Isup2.hkl

Additional supplementary materials:  crystallographic information; 3D view; checkCIF report

## Figures and Tables

**Table 1:** Hydrogen-bond geometry (Å, °)

*D*—H⋯*A*	*D*—H	H⋯*A*	*D*⋯*A*	*D*—H⋯*A*
N2—H2*B*⋯O3^i^	0.88	2.06	2.9426 (12)	175
C5—H5*A*⋯O4^ii^	1.00	2.52	3.4424 (15)	153
C10—H10*B*⋯O2^iii^	0.99	2.56	3.4228 (14)	146
C11—H11*B*⋯O3^ii^	0.99	2.53	3.5026 (13)	167
C11—H11*B*⋯O1^ii^	0.99	2.53	3.1072 (14)	117
C3—H3*A*⋯O4^iv^	0.99	2.52	3.2577 (15)	131

Symmetry codes: (i) 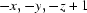; (ii) 
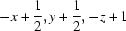
; (iii) 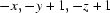; (iv) 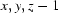.

**Fig. 1.**
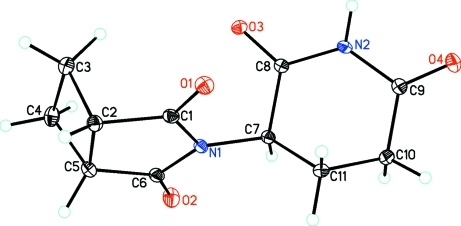
The molecular structure of C11H12N2O4, showing the atom numbering scheme and 50% probability displacement ellipsoids.

**Fig. 2.**
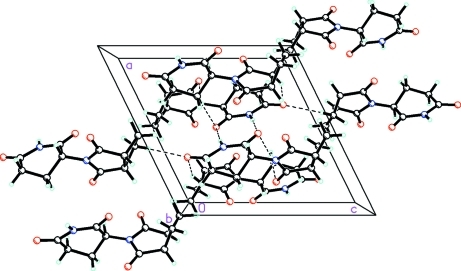
The molecular packing for C11H12N2O4 viewed down the b axis. Dashed lines indicate C–H···O and N–H···O intermolecular hydrogen bonds.

## supplementary crystallographic information

### Comment

The synthesis and biological evaluation of the title compound, 3-(2,6-dioxopiperidine-3-yl)-3-azabicyclo[3.2.0]heptane-2,4-dione and its analogues is of interest to synthetic medicinal chemists. Specifically, piperidine 2,6-dione derivatives, including those of phthalimide, are important anti-angiogenic and immunomodulative agents used for the treatment of many diseases including multiple myeloma, (Muller & Man, 2008; Yamamoto *et al.*, 2008; Zeldis, 2008), Chron's disease (Carson *et al.*,  2004), and leprosy (Werbel *et al.*, 1968). The title molecule, C_11_H_12_N_2_O_4_, a piperidine 2,6-dione derivative, consists of an azabicyclo[3.2.0]heptane group containing a nearly planar cyclobutane ring, fused to a pyrrolidine ring, bonded to a 2,6-dioxopiperidine ring at the 3 position. The six-membered dioxopiperidine ring (N2–C8–C7–C11–C10–C9) is a slightly distorted envelope, with Cremer & Pople (1975) puckering parameters Q, θ and φ of 0.5187 (12) Å, 56.12 (13)° and 176.55 (16)°, respectively. The 5-membered pyrrolidine group (N1/C2–C6) has also a slightly distorted envelope conformation with puckering parameters Q(2)and φ(2) of 0.0940 (13) Å, 82.9 (7)° respectively. For an ideal envelope θ has a value of 0 or 180° and θ(2) has a value of 72. The angle between the mean planes of the cyclobutane and fused pyrrolidine ring is 67.6 (6)° (Fig. 1). The mean planes of the pyrrolidine and cyclobutane rings make an angle of 73.9 (2)° and 62.4 (4)° with the dihedral angle of the dioxopiperidine ring, respectively. The pyrrolidine and dioxopiperidine rings are twisted about the 3-yl group [torsion angles = -55.0 (1)° (C1—N1—C7—C8) and 115.0 (1)° (C6—N1—C7—C8)] in a nearly perpendicular manner.

Crystal packing is influenced by extensive intermolecular C–H···O hydrogen bonding between all four carbonyl oxygen atoms [O1, O2, O3, O4] and hydrogen atoms from the cyclobutane (H3A & H5A) and dioxopiperidine rings (H10B & H11B) as well as by N–H···O intermolecular interactions. As a result the molecules are linked into infinite chains diagonally along the [101] plane of the unit cell in an alternate inverted pattern (Fig. 2). In addition, weak C-H··· π-ring interactions also occur between hydrogen atoms from the cyclobutane ring [H3B] and the 5-membered pyrrolidine ring [C3–H3B···*Cg*2; H3B···*Cg*2 = 2.50 Å, C3–H3B···*Cg*2 = 64°, C3···*Cg*2–H3B = 2.2475 (13) Å, *x,y,z*, where *Cg*2 = center of gravity of the N1/C1/C2/C5/C6 ring].

After a *MOPAC* AMI calculation [Austin Model 1 approximation together with the Hartree-Fock closed-shell (restricted) wavefunction was used and minimizations were terminnated at an r.m.s. gradient of less than 0.01 kJ mol^-1^ Å^-1^] with *WebMO Pro* (Schmidt & Polik, 2007), the mean planes of the cyclopropane and pyrrolidine rings became completely planar in the local minimized structure and the dihedral angle between these rings became 64.3 (8)°. The angle between the mean planes of the pyrrolidine and cyclobutane rings and the dihedral angle of the dioxopiperidine ring became 73.9 (2)° and 62.4 (4)°, respectively. The twist of the pyrrolidine and dioxopiperidine rings about the 3-yl group became more perpendicuar to each other after this geometry minimization [torsion angles = -68.6 (6)° (C1—N1—C7—C8) and 100.4 (1)° (C6—N1—C7—C8)]. Thus it is apparent that the extensive hydrogen bonding and π-ring intermolecular interactions significantly influence crystal packing for this molecule.

### Experimental

The title compound was synthesized as follows: *cis*-1,2-cyclobutane dicarboxylic acid anhydride (0.1 g, 0.79 mmol), glutamic acid (0.12 g, 0.79 mmol), DMAP (0.02 g, 0.16 mmol), and ammonium chloride (NH_4_Cl) (0.04 g, 0.916 mmol) were mixed thoroughly in a CEM-sealed vial with a magnetic stirrer. The mixture was heated for 10 min at 423 K in a CEM Discover microwave powered at 150 W. It was then cooled rapidly to 313 K and dissolved in 15 ml of (1:1) ethyl acetate: acetone. The organic layer was washed with 2x (10 ml) distilled water and dried over sodium sulfate (anhydrous). The organic layer was concentrated under vacuum and precipitated with hexanes (30 ml) affording a white solid, recrystallized from methanol, (0.10 g, 54%). mp 476–478 K; ^1^H NMR (400 MHz, DMSO-d_6_), δ (p.p.m.): 11.06 (s, 1 H, NH), 4.95 (dd, 1 H, 12.5, 5.5 Hz), 2.84 (m, 2 H), 2.52 (m, 4 H,), 2.02 (m, 2 H), 1.92 (m, 2 H); ^13^C NMR (100 MHz, DMSO-d_6_) δ (p.p.m.): 179.0(C=O), 172.7(C=O), 169.4(C=O), 49.1(CH), 37.9(CH), 37.7(CH), 30.7(CH), 22.3(CH_2_), 22.0(CH_2_), 21.0(CH_2_); MS m/*z* 236 (*M*+) 208, 151, 106, 112, 96, 83, 55, 41; IR (nujol) (ν_max_, cm^-1^): 3207.48, 1702.55, 1729.09, 1771.79 (C=O).

### Refinement

The H atoms were placed in their calculated positions and then refined using the riding model with C(N)—H = 0.88 to 1.00 Å, and with *U*_iso_(H) = 1.18–1.21*U*_eq_(C,*N*).

### Crystal data

**Table:**

C_11_H_12_N_2_O_4_	*F*(000) = 496
*M**_r_* = 236.23	*D*_x_ = 1.481 Mg m^−^^3^
Monoclinic, *P*2_1_/*a*	Mo *K*α radiation, λ = 0.71073 Å
Hall symbol: -P 2yab	Cell parameters from 4629 reflections
*a* = 10.7332 (7) Å	θ = 4.9–32.6°
*b* = 9.9358 (5) Å	µ = 0.11 mm^−^^1^
*c* = 11.0753 (7) Å	*T* = 200 K
β = 116.201 (8)°	Prism, colorless
*V* = 1059.75 (13) Å^3^	0.57 × 0.34 × 0.19 mm
*Z* = 4	

### Data collection

**Table:**

Oxford Diffraction Gemini diffractometer	3496 independent reflections
Radiation source: fine-focus sealed tube	2193 reflections with *I* > 2σ(*I*)
graphite	*R*_int_ = 0.025
Detector resolution: 10.5081 pixels mm^-1^	θ_max_ = 32.5°, θ_min_ = 4.9°
φ and ω scans	*h* = −14→16
Absorption correction: multi-scan (*CrysAlis RED*; Oxford Diffraction, 2007)	*k* = −14→13
*T*_min_ = 0.866, *T*_max_ = 0.975	*l* = −15→15
10798 measured reflections	

### Refinement

**Table:**

Refinement on *F*^2^	Primary atom site location: structure-invariant direct methods
Least-squares matrix: full	Secondary atom site location: difference Fourier map
*R*[*F*^2^ > 2σ(*F*^2^)] = 0.039	Hydrogen site location: inferred from neighbouring sites
*wR*(*F*^2^) = 0.108	H-atom parameters constrained
*S* = 0.99	*w* = 1/[σ^2^(*F*_o_^2^) + (0.0607*P*)^2^] where *P* = (*F*_o_^2^ + 2*F*_c_^2^)/3
3496 reflections	(Δ/σ)_max_ < 0.001
154 parameters	Δρ_max_ = 0.28 e Å^−^^3^
0 restraints	Δρ_min_ = −0.25 e Å^−^^3^

### Special details

**Table:**

Geometry. All e.s.d.'s (except the e.s.d. in the dihedral angle between two l.s. planes) are estimated using the full covariance matrix. The cell e.s.d.'s are taken into account individually in the estimation of e.s.d.'s in distances, angles and torsion angles; correlations between e.s.d.'s in cell parameters are only used when they are defined by crystal symmetry. An approximate (isotropic) treatment of cell e.s.d.'s is used for estimating e.s.d.'s involving l.s. planes.

### Fractional atomic coordinates and isotropic or equivalent isotropic displacement parameters (Å^2^)

**Table:**

	*x*	*y*	*z*	*U*_iso_*/*U*_eq_	
O1	0.32277 (8)	0.13630 (8)	0.42230 (7)	0.0271 (2)	
O2	0.04309 (9)	0.50429 (9)	0.26752 (8)	0.0372 (2)	
O3	0.02024 (7)	0.12103 (8)	0.39189 (7)	0.02458 (19)	
O4	0.16813 (8)	0.16581 (9)	0.83857 (8)	0.0350 (2)	
N1	0.18201 (8)	0.32243 (9)	0.37104 (8)	0.0196 (2)	
N2	0.10433 (9)	0.14479 (9)	0.61681 (8)	0.0222 (2)	
H2B	0.0683	0.0660	0.6195	0.027*	
C1	0.25634 (10)	0.22276 (11)	0.34235 (10)	0.0211 (2)	
C2	0.23146 (11)	0.24120 (12)	0.19918 (11)	0.0258 (3)	
H2A	0.3154	0.2315	0.1827	0.031*	
C3	0.09860 (12)	0.16602 (13)	0.09668 (11)	0.0340 (3)	
H3A	0.1154	0.1076	0.0330	0.041*	
H3B	0.0487	0.1164	0.1396	0.041*	
C4	0.03228 (13)	0.30239 (14)	0.03642 (11)	0.0357 (3)	
H4A	−0.0594	0.3172	0.0355	0.043*	
H4B	0.0281	0.3205	−0.0532	0.043*	
C5	0.15349 (12)	0.37539 (12)	0.15340 (11)	0.0278 (3)	
H5A	0.2019	0.4464	0.1259	0.033*	
C6	0.11702 (11)	0.41346 (11)	0.26561 (10)	0.0240 (2)	
C7	0.15765 (10)	0.31947 (11)	0.49000 (10)	0.0190 (2)	
H7A	0.0905	0.3931	0.4805	0.023*	
C8	0.08854 (9)	0.18701 (11)	0.49330 (10)	0.0188 (2)	
C9	0.17118 (10)	0.21274 (12)	0.73885 (10)	0.0234 (2)	
C10	0.24295 (11)	0.34041 (11)	0.73556 (10)	0.0249 (2)	
H10A	0.3260	0.3520	0.8228	0.030*	
H10B	0.1799	0.4172	0.7239	0.030*	
C11	0.28713 (10)	0.34228 (11)	0.62276 (10)	0.0220 (2)	
H11A	0.3561	0.2704	0.6369	0.026*	
H11B	0.3300	0.4300	0.6209	0.026*	

### Atomic displacement parameters (Å^2^)

**Table:**

	*U*^11^	*U*^22^	*U*^33^	*U*^12^	*U*^13^	*U*^23^
O1	0.0272 (4)	0.0236 (4)	0.0302 (4)	0.0056 (3)	0.0125 (3)	0.0028 (3)
O2	0.0488 (5)	0.0302 (5)	0.0331 (4)	0.0158 (4)	0.0185 (4)	0.0085 (4)
O3	0.0235 (4)	0.0233 (4)	0.0256 (4)	−0.0044 (3)	0.0096 (3)	−0.0028 (3)
O4	0.0448 (5)	0.0383 (5)	0.0276 (4)	−0.0018 (4)	0.0211 (4)	0.0041 (4)
N1	0.0226 (4)	0.0176 (5)	0.0209 (4)	0.0009 (3)	0.0117 (3)	0.0022 (4)
N2	0.0252 (4)	0.0186 (5)	0.0252 (4)	−0.0048 (3)	0.0134 (4)	0.0012 (4)
C1	0.0196 (5)	0.0198 (5)	0.0263 (5)	−0.0028 (4)	0.0124 (4)	−0.0009 (4)
C2	0.0295 (6)	0.0251 (6)	0.0279 (5)	−0.0016 (4)	0.0174 (5)	−0.0016 (5)
C3	0.0441 (7)	0.0330 (7)	0.0254 (6)	−0.0064 (5)	0.0158 (5)	−0.0041 (5)
C4	0.0385 (7)	0.0436 (8)	0.0218 (5)	0.0015 (6)	0.0103 (5)	0.0005 (5)
C5	0.0347 (6)	0.0256 (6)	0.0255 (5)	−0.0027 (5)	0.0155 (5)	0.0034 (5)
C6	0.0265 (5)	0.0203 (6)	0.0238 (5)	−0.0007 (4)	0.0098 (4)	0.0032 (4)
C7	0.0203 (5)	0.0170 (5)	0.0217 (5)	0.0004 (4)	0.0110 (4)	0.0008 (4)
C8	0.0156 (4)	0.0195 (5)	0.0224 (5)	0.0016 (4)	0.0093 (4)	0.0013 (4)
C9	0.0226 (5)	0.0247 (6)	0.0248 (5)	0.0025 (4)	0.0122 (4)	0.0012 (5)
C10	0.0294 (5)	0.0217 (6)	0.0225 (5)	−0.0019 (4)	0.0105 (4)	−0.0023 (4)
C11	0.0215 (5)	0.0193 (5)	0.0248 (5)	−0.0030 (4)	0.0099 (4)	−0.0014 (4)

### Geometric parameters (Å, °)

**Table:**

O1—C1	1.2136 (13)	C3—H3B	0.9900
O2—C6	1.2080 (13)	C4—C5	1.5520 (16)
O3—C8	1.2253 (12)	C4—H4A	0.9900
O4—C9	1.2125 (13)	C4—H4B	0.9900
N1—C1	1.3936 (14)	C5—C6	1.5069 (16)
N1—C6	1.3959 (13)	C5—H5A	1.0000
N1—C7	1.4523 (13)	C7—C8	1.5191 (15)
N2—C8	1.3679 (13)	C7—C11	1.5298 (13)
N2—C9	1.3933 (13)	C7—H7A	1.0000
N2—H2B	0.8800	C9—C10	1.4929 (16)
C1—C2	1.4984 (15)	C10—C11	1.5195 (15)
C2—C5	1.5364 (17)	C10—H10A	0.9900
C2—C3	1.5654 (15)	C10—H10B	0.9900
C2—H2A	1.0000	C11—H11A	0.9900
C3—C4	1.5390 (18)	C11—H11B	0.9900
C3—H3A	0.9900		
			
C1—N1—C6	113.25 (9)	C6—C5—H5A	115.6
C1—N1—C7	122.88 (8)	C2—C5—H5A	115.6
C6—N1—C7	123.25 (9)	C4—C5—H5A	115.6
C8—N2—C9	127.22 (9)	O2—C6—N1	123.98 (10)
C8—N2—H2B	116.4	O2—C6—C5	127.86 (10)
C9—N2—H2B	116.4	N1—C6—C5	108.14 (9)
O1—C1—N1	123.21 (9)	N1—C7—C8	108.95 (8)
O1—C1—C2	129.16 (10)	N1—C7—C11	114.71 (8)
N1—C1—C2	107.57 (9)	C8—C7—C11	110.57 (8)
C1—C2—C5	105.78 (9)	N1—C7—H7A	107.4
C1—C2—C3	112.87 (9)	C8—C7—H7A	107.4
C5—C2—C3	89.18 (8)	C11—C7—H7A	107.4
C1—C2—H2A	115.3	O3—C8—N2	120.82 (10)
C5—C2—H2A	115.3	O3—C8—C7	122.84 (9)
C3—C2—H2A	115.3	N2—C8—C7	116.33 (9)
C4—C3—C2	89.61 (9)	O4—C9—N2	119.20 (10)
C4—C3—H3A	113.7	O4—C9—C10	124.80 (10)
C2—C3—H3A	113.7	N2—C9—C10	116.00 (9)
C4—C3—H3B	113.7	C9—C10—C11	112.47 (9)
C2—C3—H3B	113.7	C9—C10—H10A	109.1
H3A—C3—H3B	111.0	C11—C10—H10A	109.1
C3—C4—C5	89.58 (8)	C9—C10—H10B	109.1
C3—C4—H4A	113.7	C11—C10—H10B	109.1
C5—C4—H4A	113.7	H10A—C10—H10B	107.8
C3—C4—H4B	113.7	C10—C11—C7	107.91 (9)
C5—C4—H4B	113.7	C10—C11—H11A	110.1
H4A—C4—H4B	111.0	C7—C11—H11A	110.1
C6—C5—C2	104.34 (9)	C10—C11—H11B	110.1
C6—C5—C4	112.24 (10)	C7—C11—H11B	110.1
C2—C5—C4	90.21 (9)	H11A—C11—H11B	108.4
			
C6—N1—C1—O1	178.13 (10)	C2—C5—C6—O2	−171.68 (11)
C7—N1—C1—O1	−10.65 (15)	C4—C5—C6—O2	−75.45 (15)
C6—N1—C1—C2	−4.49 (11)	C2—C5—C6—N1	7.10 (11)
C7—N1—C1—C2	166.72 (9)	C4—C5—C6—N1	103.32 (11)
O1—C1—C2—C5	−174.12 (11)	C1—N1—C7—C8	−55.34 (12)
N1—C1—C2—C5	8.72 (11)	C6—N1—C7—C8	115.01 (10)
O1—C1—C2—C3	89.96 (14)	C1—N1—C7—C11	69.19 (12)
N1—C1—C2—C3	−87.20 (11)	C6—N1—C7—C11	−120.47 (10)
C1—C2—C3—C4	115.83 (10)	C9—N2—C8—O3	−175.72 (9)
C5—C2—C3—C4	9.02 (9)	C9—N2—C8—C7	3.25 (15)
C2—C3—C4—C5	−8.93 (9)	N1—C7—C8—O3	−24.63 (13)
C1—C2—C5—C6	−9.47 (11)	C11—C7—C8—O3	−151.56 (9)
C3—C2—C5—C6	104.11 (9)	N1—C7—C8—N2	156.42 (9)
C1—C2—C5—C4	−122.52 (9)	C11—C7—C8—N2	29.49 (12)
C3—C2—C5—C4	−8.94 (9)	C8—N2—C9—O4	174.97 (10)
C3—C4—C5—C6	−96.51 (11)	C8—N2—C9—C10	−5.28 (15)
C3—C4—C5—C2	9.10 (9)	O4—C9—C10—C11	153.52 (11)
C1—N1—C6—O2	177.01 (10)	N2—C9—C10—C11	−26.21 (13)
C7—N1—C6—O2	5.83 (16)	C9—C10—C11—C7	56.95 (12)
C1—N1—C6—C5	−1.83 (11)	N1—C7—C11—C10	178.27 (9)
C7—N1—C6—C5	−173.01 (9)	C8—C7—C11—C10	−58.05 (11)

### Hydrogen-bond geometry (Å, °)

**Table:**

*D*—H···*A*	*D*—H	H···*A*	*D*···*A*	*D*—H···*A*
N2—H2B···O3^i^	0.88	2.06	2.9426 (12)	175
C5—H5A···O4^ii^	1.00	2.52	3.4424 (15)	153
C10—H10B···O2^iii^	0.99	2.56	3.4228 (14)	146
C11—H11B···O3^ii^	0.99	2.53	3.5026 (13)	167
C11—H11B···O1^ii^	0.99	2.53	3.1072 (14)	117
C3—H3A···O4^iv^	0.99	2.52	3.2577 (15)	131

Symmetry codes: (i) −*x*, −*y*, −*z*+1; (ii) −*x*+1/2, *y*+1/2, −*z*+1; (iii) −*x*, −*y*+1, −*z*+1; (iv) *x*, *y*, *z*−1.
